# Sorafenib increases 18-FDG colic uptake: demonstration in patients with differentiated thyroid cancer

**DOI:** 10.1186/2191-219X-2-18

**Published:** 2012-05-07

**Authors:** Renaud Ciappuccini, Géraldine Trzepla, Natacha Heutte, Emmanuel Sevin, Marie Pierre Galais, Stéphane Bardet

**Affiliations:** 1 Departments of Nuclear Medicine and Thyroid Unit, Centre François Baclesse, 3 Avenue Général Harris – BP 5026, Cedex 05, Caen, 14076, France; 2 Department of Endocrinology, Centre Hospitalo-Universitaire, Caen, 14000, France; 3 GRECAN EA-1772, Université de Caen-Basse Normandie, and Department of Clinical Research, Centre François Baclesse, Caen, 14076, France; 4 Department of Oncology, Centre François Baclesse, Caen, 14076, France

**Keywords:** Sorafenib, FDG PET, Thyroid cancer, Colic uptake

## Abstract

**Background:**

To assess 18-fluorodeoxyglucose (FDG) bowel uptake in patients with differentiated thyroid cancer (DTC) treated with sorafenib.

**Findings:**

Visual (5-point scale) and high maximum standard uptake value (SUVmax) semi-quantitative analyses were conducted in 63 positron emission tomography (PET) studies performed in patients on sorafenib (group 1, *n* = 20), in a control group (group 2, *n* = 28) and in patients on sunitinib or vandetanib (group 3, *n* = 15).

Moderate or high and diffuse bowel uptake (grade 4 or 5) was observed in 90% of the PET scans of group 1 versus none in group 2. Only 20% of PET scans in group 3 were scored grade 4. SUVmax values were significantly higher for all colic segments in group 1 than in group 2 (*P* < 0.0001) or 3 (*P* < 0.0004). This uptake pattern appeared rapidly (one month) and disappeared after sorafenib withdrawal.

**Conclusions:**

FDG uptake is increased in the colon of DTC patients treated by sorafenib.

## Findings

### Background

Sorafenib is a new targeted therapy with an angiogenesis inhibiting activity, belonging to the tyrosine kinase inhibitors (TKI) family [1]. This multikinase inhibitor has been evaluated in various cancers with promising results [2-4], including in medullary [5] and in differentiated thyroid cancer (DTC) [6,7]. In the first patients with DTC treated by sorafenib in our unit, we often noticed an intense and diffuse 18-Fluorodeoxyglucose (FDG) uptake in the abdomen. The aim of the present retrospective work was to assess the proportion of patients with such an uptake, to determine the uptake pattern in the small intestine and in the colon, to evaluate the scintigraphic outcome pattern after sorafenib withdrawal when possible and to compare this uptake with that of patients treated by other TKI such as sunitinib and vandetanib.

## Methods

### Patients

Between June 2008 and July 2011, 169 FDG PET/CT studies were performed in 85 patients with DTC. Among these, 61 PET/CT studies were selected for analysis and pooled in the three following groups. Group 1 included 20 PET/CT studies performed in six patients with radioiodine negative progressive metastatic disease treated by sorafenib. The control group (group 2) included 26 PET/CT studies performed in 18 of the 75 DTC patients never treated by TKIs nor metformin, matching with patients of group 1 for sex, age and weight. Group 3 included 15 PET/CT studies in five patients treated by sunitinib or vandetanib.

### 18-FDG PET/CT acquisition

Patients were injected intravenously with 265 MBq (range, 177–410) of 18-FDG. PET images were acquired from the mid-thigh to the skull, 60 min after injection. PET/CT scans were performed using a combined PET/CT scanner (Biograph 6, Siemens Medical Solutions, Malvern, PA, USA).

### Data analysis

The interpretation of PET/CT scans was performed by two experienced nuclear medicine physicians (SB, RC) who were unaware of the group assignment. Images were reviewed for visual and semi-quantitative analyses on a Leo workstation (Siemens Medical Solutions, Malvern, PA, USA). Both physicians worked separately. When discrepancies occurred, studies were reviewed together to achieve a consensus.

For visual analysis, the gastrointestinal tract uptake was scored on maximum intensity projection images by using a 5-point scale adapted from Gontier et al. [8]. Grade 1 corresponded to an activity lower than the hepatic background, grade 2 to an activity similar to that of the liver, grade 3 to an uptake involving one or two colic segments with an intensity moderately higher than the hepatic activity, grade 4 to a diffuse uptake with an intensity moderately higher than the liver and grade 5 to an intense and diffuse uptake.

For semi-quantitative analysis, three high maximum standard uptake value (SUVmax) measurements were performed on the small bowel segments (third duodenum, jejunum and distal ileum loop) and five on the colic segments (caecum, ascending colon, transverse colon, descending colon, sigmoid colon). SUVmax values were calculated by drawing regions of interest (1-cm diameter circle) on PET/CT transverse slices as recommended [9].

### Statistical analysis

For visual analysis, the 5-point scale assignments were compared in the three groups using the Fisher exact test. For semi-quantitative analysis, SUVmax values were compared using the nonparametric Kruskal-Wallis test, and if necessary, using the Wilcoxon test. All tests were two-sided. Because of the limited number of studies in each group, differences were considered statistically significant if *P* < 0.001. Statistical Analysis Software 9.2 (SAS Institute Inc., Cary, NC, USA) was used for data analysis.

## Results

Characteristics of patients are reported in Table 1. No patients had metformin intake. Patients of groups 1 to 3 were similar for age, sex, weight and body mass index. Five out of six patients of group 1 and none in group 3 presented grade 1 diarrhoea. Three patients of group 1 were given loperamide when needed.

**Table 1:** Characteristics of patients

	**Group 1**	**Group 2**	**Group 3**
FDG PET/CT scans (*n*)	20	28	15
Patients (*n*)	6	18	5
Men/women	3/3	9/9	2/3
Age (years)	67 ± 10	74 ± 9	65 ± 13
Weight (kilograms)	72 ± 10	67 ± 10	67 ± 10
Body Mass Index (kilogram/square meter)	25.7 ± 5.2	25.0 ± 3.8	24.2 ± 4.1
Histology			
Papillary	3	13	2
Follicular	2	4	1
Poorly differentiated	1	1	2
PET on TKI (*n*)			
Sorafenib	20	0	0
Sunitinib	0	0	4
Vandetanib	0	0	11
Patient status (*n*)			
On suppressive LT4 therapy	20	23	15
Off LT4	0	2	0
After recombinant human thyrotropin	0	3	0

*LT4* levothyroxine.

### PET/CT data: visual analysis

**Figure 1:**
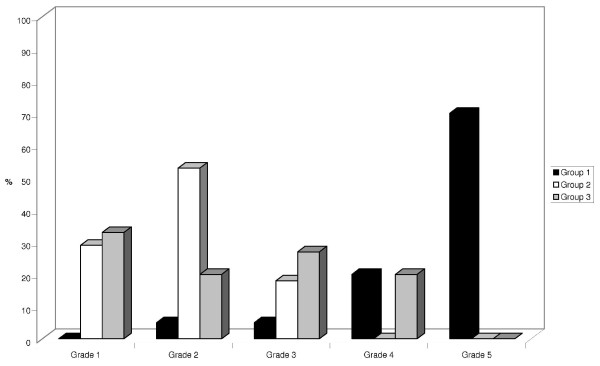
**Visual analysis in patients treated by sorafenib.** Visual analysis in patients treated by sorafenib (group 1, *black*), in control patients (group 2, *white*) and in patients treated by vandetanib or sunitinib (group 3, *grey*).

In group 1, 90% of PET scans were scored grade 4 (20%) or grade 5 (70%), whereas no PET was scored grade 4 or 5 in group 2 (Figure 1). In group 3, no study was scored grade 5 but three (20%) were grade 4, all on vandetanib treatment. Overall, the proportions of grade 4 and 5 were significantly higher in group 1 than in group 2 and in group 3 (*P* < 0.0001).

### PET/CT data: semi-quantitative analysis

**Figure 2:**
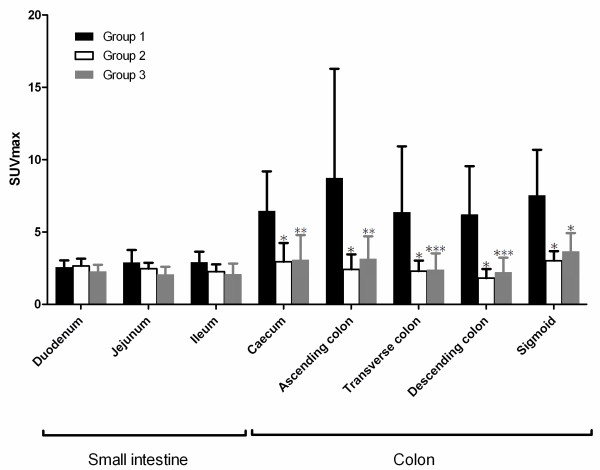
**SUVmax values in patients treated by sorafenib.** *vs. group 1, *P* < 0.0001; **vs. group 1, *P* = 0.0001; ***vs. group 1, *P* = 0.0004. SUVmax values in patients treated by sorafenib (group 1, *black*), in control patients (group 2, *white*) and in patients treated by vandetanib or sunitinib (group 3, *grey*). *Bars* denote standard deviation.

FDG uptake was significantly increased in all colic segments of group 1 as compared to group 2 (*P* < 0.0001) or group 3 (*P* < 0.0004) (Figure 2). No SUVmax differences were observed between the three groups for each segment of the small intestine.

### Outcome of FDG uptake in patients of group 1

**Figure 3:**
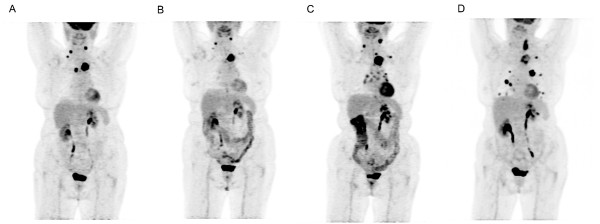
**Uptake pattern in one patient of Group 1.** Uptake pattern in one patient of Group 1 before **(A)**, during **(B, C)** and after**(D)** sorafenib treatment. The high and diffuse colic uptake (grade 5) is observed only in panels B and C.

PET/CT studies before, during and after sorafenib treatment were available in three patients, but only before and during treatment in the three other patients. The first PET/CT study on sorafenib was performed after 1 month of treatment in three patients and after 3 months in the three other ones. A grade 4 and 5 uptake appears rapidly after sorafenib introduction in all patients, including in the one who did not exhibit diarrhoea. The colic uptake pattern always disappeared after sorafenib withdrawal. Figure 3 illustrates the typical uptake outcome in one patient before, during and after sorafenib treatment.

Uptake pattern in one patient of Group 1 before (**A**), during (**B**, **C**) and after (**D**) sorafenib treatment. The high and diffuse colic uptake (grade 5) is observed only in panels B and C

## Discussion

This study shows that all patients with DTC treated by sorafenib in our experience exhibited a diffuse and intense 18-FDG intestinal uptake whereas no controls presented such a pattern. The semi-quantitative analysis demonstrates that this diffuse bowel uptake was increased only in the colon, but not in the small intestine. This typical pattern rapidly appears after sorafenib introduction, persists during treatment and disappears after withdrawal. In contrast, although digestive side effects of sunitinib and vandetanib are quite similar to those of sorafenib, only a minority of PET studies performed in patients treated by the two former showed grade 4 uptake.

To our knowledge, this typical bowel uptake during sorafenib treatment has not been described so far in previous reports in patients with DTC [10] or with other malignancies [11,12]. A quite similar uptake pattern involving both colon and small intestine has been previously described in diabetic patients on metformin treatment with unclear mechanisms [8]. What could be the hypotheses for this bowel uptake under sorafenib? Diarrhoea is a frequent side effect of sorafenib treatment with unknown origin. Motility diarrhoea might be associated with an increased uptake of the smooth intestinal muscles. One patient of our series, however, presented increased bowel uptake without diarrhoea, suggesting that other mechanisms may be involved. Although no patients had colitis-related symptoms, an inflammatory mechanism cannot be ruled out. A case of colitis which worsened after sorafenib treatment has been previously reported [13]. A vascular origin has also been hypothesized in a case of radiotherapy-induced bowel perforation under sorafenib [14]. Endoscopic and pathological data might have been relevant to better understand the mechanisms. However, such explorations were not clinically and ethically justified in our patients, all the more because diarrhoea and bowel uptake stopped after sorafenib withdrawal.

## Conclusion

FDG uptake is increased in the colon of DTC patients during sorafenib treatment. Further preclinical and clinical studies are needed to better understand the mechanisms of this uptake pattern.

## Competing interests

The authors declare that they have no competing interests.

## Authors' contributions

All authors contributed substantially to the scientific process leading to this manuscript. RC, GT and SB contributed to the concept and design of the study, data analysis, data interpretation, drafted and revised the manuscript. NH contributed to data analysis. MPG and ES critically contributed to the manuscript. All authors have read and approved the final manuscript.
